# A Privacy and Energy-Aware Federated Framework for Human Activity Recognition

**DOI:** 10.3390/s23239339

**Published:** 2023-11-22

**Authors:** Ahsan Raza Khan, Habib Ullah Manzoor, Fahad Ayaz, Muhammad Ali Imran, Ahmed Zoha

**Affiliations:** 1 James Watt School of Engineering, University of Glasgow, Glasgow G12 8QQ, UK; a.khan.9@research.gla.ac.uk (A.R.K.); h.manzoor.1@research.gla.ac.uk (H.U.M.); f.ayaz.1@research.gla.ac.uk (F.A.); muhammad.imran@glasgow.ac.uk (M.A.I.); 2 FSD-Campus, University of Engineering and Technology, Lahore 38000, Pakistan

**Keywords:** human activity recognition, wearable sensing, LSTM, CNN, spiking neural network, federated learning

## Abstract

Human activity recognition (HAR) using wearable sensors enables continuous monitoring for healthcare applications. However, the conventional centralised training of deep learning models on sensor data poses challenges related to privacy, communication costs, and on-device efficiency. This paper proposes a federated learning framework integrating spiking neural networks (SNNs) with long short-term memory (LSTM) networks for energy-efficient and privacy-preserving HAR. The hybrid spiking-LSTM (S-LSTM) model synergistically combines the event-driven efficiency of SNNs and the sequence modelling capability of LSTMs. The model is trained using surrogate gradient learning and backpropagation through time, enabling fully supervised end-to-end learning. Extensive evaluations of two public datasets demonstrate that the proposed approach outperforms LSTM, CNN, and S-CNN models in accuracy and energy efficiency. For instance, the proposed S-LSTM achieved an accuracy of 97.36% and 89.69% for indoor and outdoor scenarios, respectively. Furthermore, the results also showed a significant improvement in energy efficiency of 32.30%, compared to simple LSTM. Additionally, we highlight the significance of personalisation in HAR, where fine-tuning with local data enhances model accuracy by up to 9% for individual users.

## 1. Introduction

Human activity recognition (HAR) aims to identify the physical movements of people, enabling intelligent systems to assist individuals in improving their quality of life with applications spanning smart homes, healthcare, public safety, and sports [1]. The rapid proliferation of wearable devices, such as smartphones and smartwatches, has fuelled advancements in HAR by providing rich contextual data for applications like remote patient monitoring, sports, and lifestyle management [2]. These advances are particularly salient in elderly care, where HAR can facilitate independence and timely interventions, mitigating risks such as falls. Despite these advances, HAR faces critical challenges, including accuracy, real-time processing, data scarcity, computational complexity, and, notably, user privacy.

Building on the importance of HAR, especially in the context of independent living for the elderly, two primary technologies have emerged for data acquisition: vision-based and sensor-based systems. Vision sensing relies on high-resolution cameras coupled with advanced computer vision techniques. However, it faces challenges such as privacy concerns and quality degradation due to lighting conditions and camera limitations [3]. In contrast, sensor-based techniques offer both wearable and non-wearable solutions. Non-wearable sensors, especially those utilising radio frequencies (RFs), like channel state information (CSI) or received signal strength indicator (RSSI), are gaining popularity for indoor human activity monitoring because of their non-invasive and privacy-conscious nature [4,5,6]. Wearable sensors, including pedometers, accelerometers, and gyroscopes, remain popular choices for HAR, with smartphones and smartwatches emerging as preferred devices for activity tracking.

The decision to use vision- or sensor-based systems depends on application requirements, environment, and user preferences, each with its own advantages and challenges. However, wearable sensors provide more accurate data for HAR, as they directly capture detailed human movements. This precision is crucial, especially in dynamic environments, and is particularly vital for elderly care, where individuals may engage in activities that vary in intensity and nature [7]. On the other hand, non-invasive sensing techniques, such as video, present significant privacy concerns, making them less suitable for applications where user privacy is paramount. Moreover, establishing infrastructure for outdoor HAR using vision or RF sensing presents challenges, often due to environmental factors, equipment costs, and maintenance requirements. Additionally, non-invasive RF-based systems, while promising, still face hurdles in achieving high accuracy, especially when monitoring multiple individuals simultaneously [8]. The decision between these techniques requires a careful balance of accuracy, privacy, and feasibility based on the specific context of the application.

**Figure 1:**
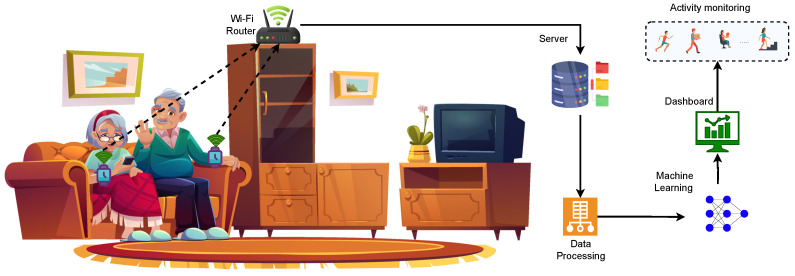
Conceptual framework of centralised indoor HAR using wearable sensors.

Traditionally, HAR systems are predominantly operated in a centralised architecture, as depicted in Figure 1. In such setups, various sensors collect data from multiple participants and share them with a central server or cloud infrastructure for processing and analysis [9]. This centralised data processing inherits several limitations, especially when the volume and variety of data sources have expanded. With the advent of advanced data analytics, deep learning (DL) has emerged as a powerful tool for HAR, enabling the extraction of intricate patterns directly from raw sensor data, thereby eliminating the need for manual feature engineering. DL models, such as convolutional neural networks (CNNs) [10] and recurrent neural networks (RNNs) [11,12], have shown remarkable success in HAR applications, often outperforming traditional machine learning techniques. However, the adoption of DL approaches in HAR is not without challenges. One of the primary concerns is data scarcity, especially labelled data, which are crucial for training DL models. The process of labelling vast numbers of data is labour-intensive and often requires domain expertise [3]. Furthermore, the centralised nature of HAR systems poses significant communication and storage costs, especially when transmitting high-dimensional raw data [13]. Additionally, processing this data in centralised servers can incur additional latency, especially when dealing with real-time activity recognition tasks. More critically, centralising user data exposes individuals to potential privacy breaches, a concern that has gained prominence in the age of stringent data protection regulations [14].

To overcome the limitations of centralised model training, federated learning (FL) has emerged as a promising distributed learning paradigm. FL enables collaborative learning using multiple participants for model training without any data sharing [15]. This distributed learning architecture offers privacy by design, reduces communication and storage overhead, and ensures real-time processing, a crucial requirement for HAR tasks [9]. Furthermore, the participation of multiple clients in FL offers significant advantages. Each participant, with their distinct data, contributes to the overall model, resulting in a more generalised and robust global model that captures a broader range of human activities. FL enables real-time processing by dividing computational tasks among different devices. Its decentralised architecture is scalable and can accommodate various devices and data sources. Additionally, FL’s collaborative training approach allows personalisation, which is achieved by fine-tuning the global model using local data, improving the accuracy and relevance of activity recognition. These personalised models use individual-specific data to provide more precise and context-aware activity recognition, aligning the system’s predictions with the user’s unique patterns and behaviours.

In the realm of HAR, the need for distributed learning is becoming increasingly important, especially given the challenges associated with centralised systems. As we transition towards more decentralised and edge-based processing, the computational demands of traditional DL models can become a significant bottleneck, especially on resource-constrained edge devices [16]. While FL offers several advantages, one major drawback is the hardware available in the market often struggles to support this distributed intelligence with energy efficiency. To overcome this challenge, neuromorphic computing emerges as a potential solution. Inspired by the human biological neural systems, neuromorphic computing promises energy-efficient and rapid signal processing. Spiking neural networks (SNNs), a subset of neuromorphic learning, are gaining attention because of their unique event-driven processing of binary inputs, known as ’spikes’ [17]. Unlike traditional DL models, SNNs operate on a temporal, event-driven paradigm, making them particularly suitable for on-device learning. The real-time and continuous nature of activity data in HAR accentuates the potential advantages of neuromorphic computing, highlighting the necessity for models that can adeptly capture the temporal dynamics of human activities.

Although SNNs are computationally efficient, traditional DL models, such as LSTM networks, are more effective in processing sequential data [18]. Given these considerations, a compelling need emerges for a model that combines the strengths of SNNs and LSTMs synergistically. Therefore, we introduce the hybrid neuromorphic federated learning (HNFL) approach, which integrates the SNN with LSTM, creating a spiking-LSTM (S-LSTM) model. The S-LSTM is ingeniously crafted to leverage the computational efficiency of SNNs while harnessing the sequential data processing capabilities of LSTMs. This fusion offers a harmonious balance between efficiency and accuracy, positioning the S-LSTM as a pioneering model for HAR in a federated setting. To the best of the authors’ knowledge, no prior research has presented such a hybrid model for HAR. The key contributions of this paper are as follows:

- We introduce a novel HNFL framework tailored for HAR using wearable sensing technology. The hybrid design of S-LSTM integrates the strengths of both LSTM and SNN seamlessly in a federated setting, offering privacy preservation and computational efficiency.
- A comprehensive analysis is conducted using two distinct publicly available datasets, and the results of the S-LSTM are compared with LSTM, spiking CNN (S-CNN), and simple CNN. This dual-dataset testing approach validates the robustness of the proposed framework and provides valuable insights into its performance in varied environments and scenarios.
- This study addresses a significant issue of client selection within the context of federated HAR applications. We conduct a thorough investigation into the implications of random client selection and its impact on the overall performance of the HAR model. This analysis provides valuable insights into achieving the optimal balance between computation, communication efficiency and model precision, which guides the ideal approach for client selection in federated scenarios.

The rest of the paper is organised as follows: Section 2 introduces the related work for FL-based HAR. In Section 3, preliminaries and the system model are discussed, whereas Section 4 explains the simulation setup. Section 5 provides the details on results and discussion, and Section 6 concludes the research findings.

## 2. Related Work

HAR has witnessed significant advancements, particularly with the proliferation of wearable sensors across diverse applications. Initially dominated by traditional machine learning techniques, HAR has shifted towards more sophisticated DL models, offering enhanced accuracy and reliability. However, with the digital landscape becoming increasingly decentralised and privacy-focused, it is imperative to adapt these models to ensure user privacy while maintaining computational efficiency. Neuromorphic computing, particularly the SNN, emerges as a promising solution to overcome these challenges. Hence, this section reviews the state-of-the-art work in (a) a centralised learning-based HAR system and (b) a federated learning-based HAR.

### 2.1. Centralised Learning-Based HAR Systems

In recent times, various studies have investigated HAR using wearable sensing and DL. For instance, the authors in [19] proposed a novel heterogeneous convolution operation that was explicitly introduced for HAR. This approach divides convolutional filters into two uneven groups. The smaller group of filters undergoes a down-sampling process and captures the broader perspective from input samples. The output of this process provides feedback to the larger group of filters, recalibrating the model to enhance feature diversity. This method demonstrated significant performance improvements through extensive testing on multiple HAR datasets without necessitating changes to the underlying network architecture. Notably, the heterogeneous convolution can be seamlessly integrated into standard convolutional layers without adding computational overhead. In [20], a novel approach that uses a DL method called neural structured learning was proposed. This system used LSTM to process wearable sensor data and applied non-linear generalised discriminant analysis to extract features. The analysis showed a high recall rate of 99% on a publicly available dataset, surpassing traditional methods, like CNN and RNN, which only achieved a maximum recall rate of 94%. In [21], a hybrid approach was introduced for HAR, leveraging a bi-convolutional recurrent neural network (Bi-CRNN) for feature extraction. The proposed scheme employed a random forest classifier for final predictions. This approach, enhanced by an auto-fusion technique for multi-sensor data processing, outperformed existing HAR algorithms. Despite its computational demands, the feature extraction ability of Bi-CRNN significantly improved its performance, achieving a remarkable 94.7% accuracy.

The study in [22] proposed a fast and robust deep convolutional neural network (FR-DCNN) for HAR using smartphone data. This framework optimised computational efficiency by employing a data compression module, allowing for swift training while maintaining high precision in recognising 12 distinct activities. The FR-DCNN model used raw data samples collected from triaxial accelerometers and gyroscopes. Notably, its performance surpassed other DL algorithms in terms of speed and accuracy, achieving a prediction accuracy of 95.27%. Even with compressed datasets, the accuracy remained impressive at 94.18%. Another innovative approach is the merging-squeeze-excitation (MSE) technique for HAR using wearable sensors [23]. This method recalibrates feature maps during fusion, allowing the model to emphasise or suppress certain features based on their relevance. This recalibration, combined with local and global skip connections, global channel attention, and deeper fusion, enhances the model’s adaptability and precision. When tested with DL models like LeNet5, AlexNet, and VGG16 for feature extraction, the proposed methods consistently achieved high accuracy across three different datasets. In summary, the current landscape of DL for HAR involves the combination of novel architectures, hybrid models, and advanced feature recalibration techniques. These advancements collectively aim to enhance the accuracy, reliability, and adaptability of HAR systems, making them more suited for real-world applications.

### 2.2. Federated Learning-Based HAR

With the evolution of the digital landscape, user privacy and data decentralisation are becoming more important. Hence, FL offers a paradigm shift from traditional centralised training, allowing models to be trained directly on an edge node (participant) without transferring raw data to a central server. This preserves user privacy and reduces communication overheads, making it suitable for HAR applications. In recent years, numerous studies have investigated FL-based HAR. For instance, the authors in [13] proposed a multimodal data fusion approach for fall detection in an FL environment. The time-series data from wearable sensors are initially transformed into images using the gramian angular field method. The fusion process combines the transformed data with visual data captured using cameras. This input-level fusion enhanced fall detection accuracy, achieving 99.92% for binary fall detection and 89.76% for multi-class fall activity recognition. The study in [3] introduced FedHAR, a personalised framework designed to deal with privacy, label scarcity, and real-time processing using smartphone and wearable sensing data. Furthermore, a novel algorithm was proposed for computing unsupervised gradients and an aggregation strategy to handle concept drift and convergence instability in online learning. Experimental results from two real-world HAR datasets show that FedHAR outperformed existing methods. Notably, when customised for unlabelled clients, it achieved an average improvement of around 10% across all metrics.

In real-world environments, HAR systems often face challenges due to non-independently and -identically distributed (Non-IID) data, in which the data distribution varies across devices, leading to imbalances and inconsistencies. This can severely impact the performance of HAR systems, as they may become biased towards specific activities or users, reducing their generalisation capability across diverse real-world scenarios. Hence, the study in [24] proposed ProtoHAR, a prototype-guided FL framework designed for sensor-based HAR, to deal with Non-IID data. ProtoHAR addressed this issue by decoupling representation and classifiers, using a global activity prototype to correct local representations and optimising user-specific classifiers for personalised HAR. This ensured privacy and reduced local model drift during customised training. The study also showed that ProtoHAR outperformed existing FL methods in terms of accuracy and convergence speed when tested on four publicly HAR datasets. However, it is important to note that the current ProtoHAR model assumes a static activity data distribution and cannot continuously learn from new data without retraining. The study in [25] introduced ClusterFL, a novel clustering-based FL system tailored for HAR. This approach was designed to understand the intrinsic relationships among data from different users. This framework minimised training loss across multiple models while intuitively identifying clustering relationships among nodes, allowing it to exclude slower or less-correlated participants within each cluster efficiently and, hence, leading to faster convergence without sacrificing accuracy and reducing communication overhead by 50%. Similarly, a novel federated learning via augmented knowledge distillation (FedAKD) was designed for the collaborative training of heterogeneous DL models [26]. FedAKD showed significant communication efficiency, surpassing the federated averaging (FedAvg) algorithm by 200 times. Additionally, it achieved 20% higher accuracy than other knowledge distillation–based FL methods. In summary, FL has emerged as a revolutionary approach to HAR. The studies highlighted in the literature underscore the versatility of FL in handling Non-IID data distributions and optimising communication efficiency in various scenarios.

As the digital landscape evolves, demand for on-device processing is increasing. However, conventional DL algorithms often struggle to run efficiently on edge devices because of computational demands, particularly in real-time processing. Neuromorphic computing, particularly SNNs, emerges as a promising solution to address this challenge. Several recent studies, including [16,27,28], have investigated the potential of SNNs. These studies have explored the use of SNNs as an alternative to traditional DL models, demonstrating how they can improve energy efficiency and accuracy in federated environments.

However, it is important to note that the HAR domain has significantly shifted from traditional machine learning to more advanced DL models and then to FL. The focus has been primarily on individual aspects, such as accuracy (in centralised systems), communication efficiency, and privacy (in FL). Furthermore, most existing studies in the literature focused on the energy efficiency of SNNs, and no study has yet harnessed the combined power of DL and SNNs, specifically for HAR. Additionally, as HAR devices continue to miniaturise, the desire for on-device processing grows, requiring an energy-efficient model. Hence, we propose a hybrid model S-LSTM that combines the computational efficiency of SNNs with the sequential data processing capabilities of LSTMs. This fusion aims to provide a balanced trade-off between the energy efficiency and accuracy of HAR using wearable sensors.

## 3. Preliminaries and System Model

This section explains the foundational principles of FL and SNNs and introduces a hybrid model, S-LSTM, that combines both paradigms to improve the performance of HAR.

### 3.1. Federated Learning

**Figure 2:**
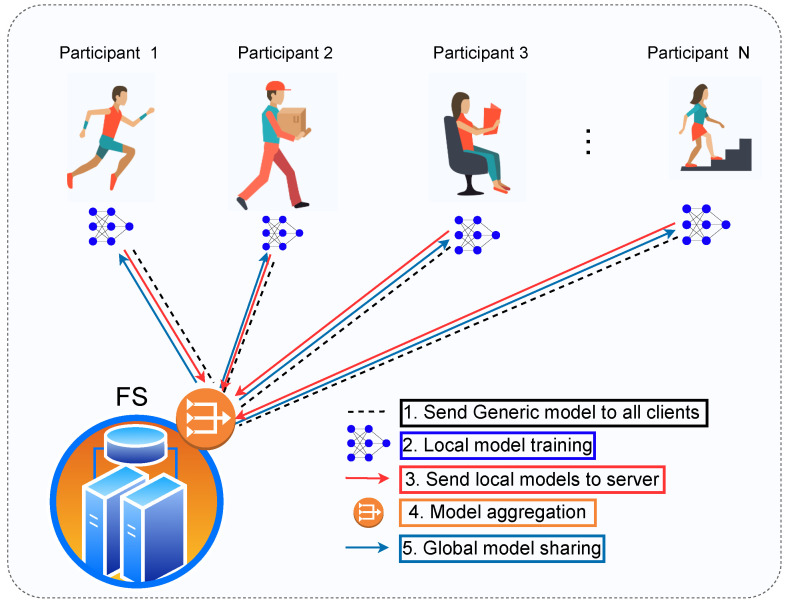
Conceptual FL framework for HAR using wearable sensing in the outdoors.

FL is a distributed learning approach that trains the model across multiple participants, where the dataset is highly decentralised. As illustrated in Figure 2, this framework consists of a federated server (FS) and *N* participants capable of processing data effectively. The FS is the controlling entity that orchestrates the model training and aggregation process. FL is an iterative process involving several communication rounds between the FS and participants to obtain an updated global model. Initially, the FS initialises and shares the global model parameters. The participants at the edge train its model using the local data and send back updates to the server. The server aggregates the global model and shares the updated model with participants. This process continues until the global model achieves the desired accuracy or maximum number of rounds reached.

For each participant i=1,…,N, there is a local copy of the dataset, which is represented by $|D^{\left(i\right)}|\equiv D$, where $D^{\left(i\right)}$ is the subset of the dataset at the $i^{\mathrm{th}}$ device and the entire dataset is given by $D=\sum_{n=1}^{N}\left|D^{\left(i\right)}\right|$. Given the model parameter vector ψ and loss function p(ψ,x) for any example *x*, the local empirical loss at the $i^{\mathrm{th}}$ participant is denoted as

(1) $$P^{\left(i\right)}\left(\psi \right)=\frac{1}{|D^{\left(i\right)}|}\sum_{x\in D^{\left(i\right)}}p\left(\psi ,x\right),$$

where $P^{\left(i\right)}\left(\psi \right)$ represents the local loss function for $i^{\mathrm{th}}$ participant based on the parameter vector ψ. Using the local loss function, FL optimises the global function on the FS mathematically represented as

(2) $$\min_{\psi }P\left(\psi \right):=\frac{1}{\sum_{i=1}^{N}\left|D^{\left(i\right)}\right|}\sum_{i=1}^{N}\left|D^{\left(i\right)}\right|P^{\left(i\right)}\left(\psi \right),$$

where P(ψ) is the global loss function based on the dataset *D*. FL is an iterative process; hence, for time iterations t=1,…,T, each participant compute local gradient using following equation:

(3) $$\psi^{\left(i\right)}\left(t\right)={\tilde{\psi }}^{\left(i\right)}\left(t-1\right)-\eta \nabla p\left({\tilde{\psi }}^{\left(i\right)}\left(t-1\right),x^{\left(i\right)}\left(t\right)\right),$$

where $\psi^{\left(i\right)}\left(t\right)$ represents model parameters at the $i^{th}$ participant, η is the learning rate, ${\tilde{\psi }}^{\left(i\right)}\left(t-1\right)$ represents the model parameters of previous iteration, and $\nabla p({\tilde{\psi }}^{\left(i\right)}\left(t-1\right),x^{\left(i\right)}$ is the gradient of loss function *p* with respect to model parameters for the data point $x^{\left(i\right)}\left(t\right)$. The FS performs the model aggregation once all the local updates are received, and this process is mathematically represented as

(4) $$\psi \left(t\right)=\frac{1}{\sum_{i=1}^{N}\left|D^{\left(i\right)}\right|}\sum_{i=1}^{N}\left|D^{\left(i\right)}\right|\psi^{\left(i\right)}\left(t\right)$$

### 3.2. Spiking Neural Network

**Figure 3:**
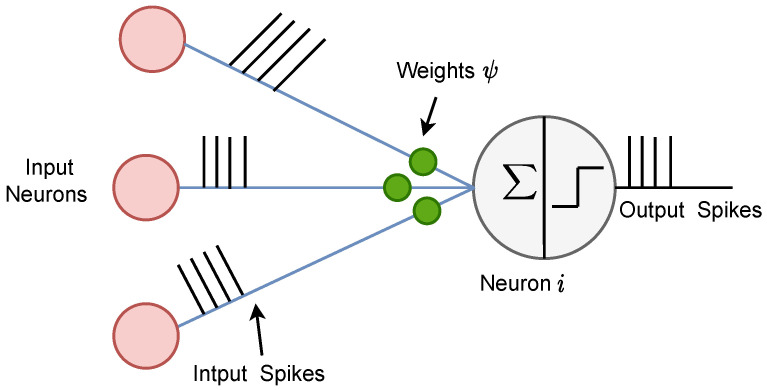
Spiking neurons propagation process.

SNNs are inspired by biological neural networks that use discrete events ’spike’ for information processing, as shown in Figure 3. SNNs offer substantial improvements in computational efficiency over traditional DL models because of their event-driven activation, sparse information coding, and lower precision arithmetic. Event-driven computing is enabled using discrete spikes for processing, allowing them to remain inactive until incoming spikes are received [16]. Additionally, at any given time, only a subset of neurons in SNN are actively spiking to achieve sparsity. Furthermore, spike signals in SNNs are binary (1s and 0s), which are processed using low-precision arithmetic. Collectively, these attributes allow SNNs to operate using significantly fewer computations per data sample, making SNNs uniquely well-suited for efficient processing on resource-constrained edge devices [29].

Each spiking neuron has a membrane potential that accumulates spike signals received from other neurons. Depending on the duration *t*, the membrane potential of a neuron can increase, decrease, or stay the same. If the potential exceeds a certain threshold voltage $v_{th}$, the neuron generates a spike signal that is transmitted to the next layer of neurons. After this, the neuron enters a short refractory phase, where its membrane potential stays constant, regardless of any incoming spikes. To simulate the spiking behaviour of neurons, we utilised a widely recognised leaky-integrate-and-fire (LIF) model because of its straightforwardness and adaptability [30]. This model is closely analogous to an electrical circuit comprising a capacitor *Q*, a resistor *Z*, a power source *V*, and an input current *J*. The LIF model in layer *l* for a neuron with index number *i* can be mathematically represented as follows [31]:

(5) $$\tau_{q}\frac{dV_{i}^{\left(l\right)}\left(t\right)}{dt}=-\left(V_{i}^{\left(l\right)}\left(t\right)-V_{res}\right)+ZJ_{i}\left(t\right),$$

\ where $\tau_{q}=Q\cdot Z$ denotes the membrane potential time constant decay, $V_{i}^{\left(l\right)}\left(t\right)$ is the neuron’s membrane potential at time *t*, *Z* is the resistance, and $J_{i}\left(t\right)$ represents the input current at time *t*, which denotes the pre-synaptic inputs. When $V_{i}^{\left(l\right)}\left(t\right)$ exceeds the given threshold $v_{th}$, the neuron generates a spike and resets its membrane voltage to $V_{res}$. Hence, for a specific layer *l* and neuron index *i*, the membrane potential $V_{i}^{\left(l\right)}$ at time step *t* can be expressed as [31,32]

(6) $$V_{i}^{\left(l\right)}\left(t\right)=\beta V_{i}^{\left(l\right)}\left(t-1\right)+\sum_{j}\psi_{ij}o_{j}^{\left(l-1\right)}\left(t\right),$$

where β (0<β<1) is the leakage factor, *j* represents the number of neurons in the previous layer l−1, $\psi_{ij}$ is the weight from neuron *j* in layer l−1 to neuron *i* in layer *l*, and $o_{j}^{\left(l-1\right)}\left(t\right)$ is the binary output of neuron *j* in layer l−1 at time *t*. The spike sequence emitted by neuron *i* in layer *l* at time *t*, when $V_{i}^{\left(l\right)}\left(t\right)$ is greater than the firing threshold $v_{th}$, can be defined as [31]

(7) $$o_{i}^{\left(l\right)}\left(t\right)=\left\{\begin{array}{cc} 1, & \mathrm{if}\;V_{i}^{\left(l\right)}\left(t\right)\geq v_{th}\\ 0, & \mathrm{otherwise} \end{array}\right.$$

In SNNs, neurons communicate through discrete spike events, where the decision is based on a step function, which is non-differentiable at the threshold. This non-differentiable behaviour poses challenges for gradient-based optimisation methods, like backpropagation, which rely on continuous and differentiable activation functions [33]. The core issue is that the step function’s gradient is either zero or undefined, preventing meaningful weight updates during training. To address this, surrogate gradient methods have been introduced, which employ a smooth, differentiable approximation of the step function during the backward pass, allowing for gradient computation. This approach ensures that the network can be trained using traditional techniques but still operate with the unique spiking behaviour of SNNs during inference. The surrogate piece-wise linear function, aligning with the previously established notation, is mathematically represented as [16,31]:

(8) $$\frac{\partial o_{i}^{\left(l\right)}\left(t\right)}{\partial V_{i}^{\left(l\right)}\left(t\right)}=\gamma \max\left(0,1-\frac{|V_{i}^{\left(l\right)}\left(t\right)-v_{th}|}{v_{th}}\right),$$

where γ is a scaling factor controlling the SNNs’ update magnitude and $v_{th}$ is the threshold voltage for spiking. In essence, the backpropagation method in SNNs mirrors that of ANNs, with the exception of using a surrogate function to approximate the non-differentiable threshold function. Hence, the weight update rule on the local participant for layer *l* at a given time is mathematically represented as [32]

(9) $$\psi_{ij}^{\left(l\right)}\left(t\right)=\psi_{ij}^{\left(l\right)}\left(t-1\right)-\eta \frac{\partial p(t)}{\partial \psi_{ij}^{\left(l\right)}\left(t-1\right)},$$

where $\psi_{ij}^{\left(l\right)}\left(t-1\right)$ represent the model parameters at t−1 for neuron *j* in layer *l* to neuron *i*.

Several training techniques have been proposed to harness the full potential of SNNs and address inherent challenges, such as the non-differentiability of spike functions. One of the unsupervised methods, spike timing–dependent plasticity (STDP), employs temporal learning principles, making it suitable for unlabelled data [34]. For tasks requiring supervised learning, the backpropagation through time (BPTT) method extends the conventional backpropagation technique to propagate errors across multiple timesteps [16]. This proves invaluable for spatiotemporal datasets, capturing their inherent time dependencies. Furthermore, surrogate gradient learning was introduced to provide a continuous differentiable approximation to the non-linear spike function, thus allowing for gradient-based optimisation techniques [16]. Similarly, reward-modulated STDP combines concepts from reinforcement learning by using rewards to modulate STDP in an episodic trial-and-error manner [28]. Our problem involves training an SNN with diverse data using supervised learning. Hence, we have developed a system that combines surrogate gradient learning with BPTT as given in [35]. This integration serves two purposes. Firstly, the spike function in SNNs is non-differentiable, which makes traditional gradient-based optimisation techniques challenging. Surrogate gradient learning offers a solution by providing a continuous, differentiable approximation of the non-linear spike function, allowing for gradient-based optimisation. Secondly, to effectively capture the temporal dependencies in HAR data, we need a training technique that can handle these sequences. BPTT extends conventional backpropagation to propagate errors across multiple time steps, minimising a loss function *p* by applying a gradient descent that propagates backwards in time. As a result, the gradient of the loss *p* with respect to the weights ψ and membrane potential is given by [35]:

(10) $$\frac{\partial p(t)}{\partial \psi_{ij}}=\sum_{t}\frac{\partial p(t)}{\partial V_{i}^{\left(l\right)}\left(t\right)}\cdot o_{j}^{\left(l-1\right)}\left(t\right),$$

(11) $$\frac{\partial p(t)}{\partial V_{i}^{\left(l\right)}\left(t\right)}=\frac{\partial p(t)}{\partial o_{i}^{\left(l\right)}\left(t\right)}\cdot \frac{\partial o_{i}^{\left(l\right)}\left(t\right)}{\partial V_{i}^{\left(l\right)}\left(t\right)}+\beta \frac{\partial p(t)}{\partial V_{i}^{\left(l\right)}\left(t+1\right)},$$

where $o_{j}^{\left(l-1\right)}\left(t\right)$ represents the spike of neuron *j* in the previous layer l−1 at time *t*, contributing to the input of neuron *i* in layer *l*.

### 3.3. Long Short-Term Memory Networks

LSTM networks are a special type of recurrent neural network (RNN) that can learn long-term dependencies in sequence prediction tasks. LSTMs address the vanishing and exploding gradient problems of traditional RNNs by employing gates that regulate information flow [36]. Each LSTM unit contains a cell and three types of gates: input, output, and forget gates. These gates manage information flow into and out of the cell, enabling the LSTM to store information over extended time periods. The operations within an LSTM unit at time *t* for a single cell can be described using the following equations [37]:

(12) $$i_{t}=\sigma \left(\psi_{xi}\cdot x_{t}+\psi_{hi}\cdot h_{t-1}+b_{i}\right)$$

(13) $$f_{t}=\sigma \left(\psi_{xf}\cdot x_{t}+\psi_{hf}\cdot h_{t-1}+b_{f}\right)$$

(14) $$o_{t}=\sigma \left(\psi_{xo}\cdot x_{t}+\psi_{ho}\cdot h_{t-1}+b_{o}\right)$$

(15) $${\tilde{C}}_{t}=\tanh\left(\psi_{xc}\cdot x_{t}+\psi_{hc}\cdot h_{t-1}+b_{C}\right)$$

(16) $$C_{t}=f_{t}*C_{t-1}+i_{t}*{\tilde{C}}_{t}$$

(17) $$h_{t}=o_{t}*\tanh\left(C_{t}\right)$$

where σ represents the sigmoid function and $i_{t}$, $f_{t}$, and $o_{t}$ are the input gate, forget gate, and output gate activations at time *t*, respectively. ${\tilde{C}}_{t}$ is the candidate memory state, $C_{t}$ is the cell state, $h_{t}$ is the hidden state of the LSTM unit, ψ denotes the weight matrices, $x_{t}$ is the input vector at time *t*, and *b* represents the bias vectors. LSTMs have the unique ability to remember and forget information in a selective manner, which makes them highly suitable for handling complex tasks involving sequential data like language processing, speech recognition, and time-series prediction. Integrating LSTMs into the proposed S-LSTM model enables the processing of temporal dependencies present in the data while leveraging the computational efficiency offered by SNNs. This results in a powerful framework that can be used for HAR applications in environments with limited resources.

### 3.4. Proposed S-LSTM Model

**Figure 4:**
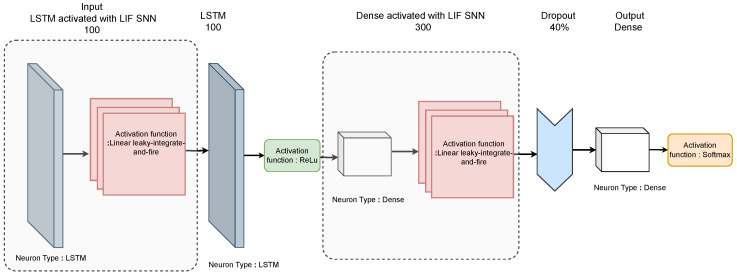
Proposed hybrid S-LSTM model where input LSTM layer activated by LIF.

Our proposed S-LSTM model seamlessly combines LSTM units with the spiking behaviour of LIF neurons, as shown in Figure 4. The model starts with an LSTM layer consisting of 100 neurons to process input data. This LSTM layer returns sequences, ensuring that the temporal dependencies in the data are captured. Next, the spiking layer with LIF replaces the conventional activation functions. The spiking layer has a trainable threshold that determines neuron firing and uses a surrogate gradient to approximate the gradient during backpropagation due to the non-differentiable nature of spiking behaviour. After this, another LSTM layer with 100 units processes the sequences further, followed by a dense layer with 300 neurons, which employs LIF neurons to replace the typical activation function. A dropout layer is added to mitigate over-fitting, followed by a fully connected output layer. The output layer uses a SoftMax activation function, producing a probability distribution over the possible activity classes. The training process of federated S-LSTM for HAR is given in Algorithm 1.
**Algorithm 1** Federated S-LSTM training with surrogate gradient and BPTT.1:**Input:** Initial model parameters $\psi_{0}$, clients i=1,2,…,N2:**Output:** Trained model parameters ψ3:**Procedure: Initialisation**4:**for** each client *i* **in parallel do**5:   $D^{\left(i\right)}\leftarrow$ local dataset6:   $\psi^{\left(i\right)}\leftarrow \psi_{0}$     {Initialise local model}7:**end for**8:**Procedure: FL training**9:**for** round r=1,2,…,R **do**10:   **for** each client i=1,2,…,N **in parallel do**11:        $\Delta \psi^{\left(i\right)}\leftarrow$LocalTraining$\left(\psi^{\left(i\right)},D^{\left(i\right)}\right)$12:   **end for**13:   $\psi_{r+1}\leftarrow$ServerUpdate$\left({\left\{\Delta \psi^{\left(i\right)}\right\}}_{i=1}^{N}\right)$     {Aggregate updates}14:   **broadcast** $\psi_{r+1}$ to clients15:**end for**16:**Procedure: LocalTraining(ψ,D)**17:Initialise local parameters $\psi^{\left(i\right)}$, learning rate η18:**for** each time step t=1,…,T **do**19:   Compute local gradient using surrogate gradient and BPTT {Based on Equations (8)–(11)}20:   Update local model parameters $\psi^{\left(i\right)}\left(t\right)$21:**end for**22:**return** model update Δψ23:**Procedure: ServerUpdate(**${\left\{\Delta \psi^{\left(i\right)}\right\}}_{i=1}^{N}$**)**24:ψ←**aggregate**($\left\{\Delta \psi^{\left(i\right)}\right\}$)     {Based on Equation (4)}25:**return** updated model parameters ψ

## 4. Simulation Setup

This section provides a detailed discussion of the datasets, performance evaluation strategy, and metrics used in this study.

### 4.1. Dataset Description

Despite HAR being a well-investigated topic, attempts to evaluate it using smartphone data is a recent and very active area of research. Several datasets have been collected using smartphones, which exhibit severe challenges, including sensor configuration, sampling frequencies, accessibility, realism, size, heterogeneity, and annotation quality. Additionally, there is an extreme class imbalance due to the stark differences in activity patterns between classes. Thus, HAR is the perfect testbed for assessing neuromorphic federated learning in practical heterogeneous contexts. Furthermore, our focus was on reproducibility, heterogeneity, and realistic datasets, which led us to select two publicly available datasets. The UCI dataset [38], which is one of the most commonly used in HAR benchmarking studies, was chosen first. However, UCI was collected in a strictly controlled laboratory environment, and the sample size was also very limited. Therefore, we also employed the Real-World dataset [39], recorded outdoors without restrictions. The details of these two datasets are explained below:

#### 4.1.1. UCI Dataset

The UCI dataset is obtained using Samsung Galaxy S II smartphones worn by 30 volunteers from distant age groups and genders. The volunteers were engaged in six daily routine activities: sitting, standing, lying down, walking, walking downstairs, and walking upstairs. Each subject repeated these activities multiple times with two distant scenarios for device placement. These scenarios included the placements of a smartphone on the left wrist and one on the preferred position of each subject. The smartphone’s embedded accelerometer and gyroscope sensors captured triaxial linear acceleration and angular velocity at a rate of 50 Hz. The raw signals were pre-processed to minimise noise interference, and 17 distinctive signals were extracted, encompassing various time and frequency domain parameters, such as magnitude, jerk, and fast Fourier transform (FFT). For analysis, signals were segmented into windows of 2.56 s, with an overlap of 50%, culminating in 561 diverse features per window derived from statistical and frequency measures. This dataset contained 10,299 instances, with a strategic split of 70% for training and 30% reserved for testing.

However, the dataset was merged and split into five subsets to make a local dataset of each participant. The data distribution among the participants was kept highly unbalanced to ensure the actual case for the FL scenario. Furthermore, the dataset of each client was further divided into training (80%) and testing (20%) splits, and the test split of each client was combined to create a global test set for performance evaluation.

#### 4.1.2. Real-World Dataset

Although the UCI dataset is very commonly used in HAR studies, it has limitations, as it was collected in a controlled laboratory environment. Additionally, the sample size was very small to explore the true potential of FL. Hence, we chose a more realistic dataset collected by Sztyler and Stuckenschmidt [39]. Data were gathered from 15 participants (eight male, seven female) executing eight common, real-world activities, including jumping, running, jogging, climbing stairs, lying, standing, sitting, and walking. The accelerometer data were collected from seven different locations on the body, which included the head, chest, upper arm, wrist, forearm, thigh, and shin. In the data collection process, smartphones and a smartwatch were mounted on the aforementioned anatomical sites, collecting the data at the frequency of 50 Hz. The dataset incorporated 1065 min of accelerometer measurements per on-body position per axis, amounting to extensive volume.

Additionally, the Real-World dataset was also well-suited for the HAR study as it is captured in a naturalistic environment, exhibiting the realistic class imbalance. For instance, the jumping activity comprised 2% of the data compared to standing, which constituted 14% of the total data. Additionally, factors such as high-class imbalance and the availability of separated user data made this dataset an appropriate choice for an extensive study on FL approaches for HAR.

### 4.2. Performance Metrics

HAR is treated as a multi-class classification problem where various metrics are used to evaluate the performance of a model. One commonly used metric is accuracy, defined as the ratio of correctly predicted instances to the total number of cases. However, the accuracy limitations become pronounced in the context of highly imbalanced datasets. For example, in scenarios where one class dominates a dataset, a model may achieve higher accuracy by simply predicting that class, disregarding the distribution of other classes. This phenomenon is known as the accuracy paradox, highlighting the risk of relying solely on accuracy as a performance metric when dealing with diverse datasets. Therefore, the alternative metrics to evaluate the performance of the model are precision, recall, and F1-score, defined as follows:

**Precision:** This metric quantifies the number of correct positive predictions made by the model relative to the total number of positive predictions mathematically represented as

(18) $$\mathrm{Precision}=\frac{\mathrm{TP}}{\mathrm{TP}+\mathrm{FP}},$$

where TP and FP are true positives and false positives, respectively.

**Recall (sensitivity):** This metric measures how well the model can correctly identify positive instances, which is particularly important in contexts where missing positive instances (false negatives) can have serious consequences. Recall is mathematically represented as

(19) $$\mathrm{Recall}=\frac{\mathrm{TP}}{\mathrm{TP}+\mathrm{FN}},$$

where FN represents a false negative.

**F1-score:** It is the harmonic mean of precision and recall, which provides a balance between the two metrics, especially when there’s an uneven class distribution. F1-score is mathematically represented as

(20) $$F1-\mathrm{score}=2\times \frac{\mathrm{Precision}\times \mathrm{Recall}}{\mathrm{Precision}+\mathrm{Recall}}$$

Furthermore, all experimental procedures were conducted in a simulated environment for a comprehensive evaluation. This allowed us to gauge the effectiveness of the models’ two metrics: global performance evaluation and personalised model assessment. The global metric was used to determine the proficiency of the model across an entire dataset using a global test set, which helped to assess its generalisation capabilities. On the other hand, personalised performance assessments were performed at the participant level, with the best global model fine-tuning using local data. This personalised training created a customised model, and its performance was evaluated using the local test set. We compared the results of both personalised and global models for each participant. Additionally, the energy efficiency of the proposed model was also evaluated, which mainly depends on the two factors, i.e., the computation time required for local training and parameter transfer during each communication round. This metric is defined as the energy estimate mathematically given as [40]

(21) $$E_{est}=R\left[\left(\alpha *t_{com}\right)+N\left(\beta *P_{trn}\right)\right],$$

where α is the computation constant having dimensions of energy per second and β is communication constant with dimension of energy per kilobytes. *R* represents the number of communication rounds; *N* is the number of participants; $t_{com}$ is the computation time, which is dependent on the device type; and $P_{trn}$ is the size of parameters transferred in each communication round.

## 5. Results and Discussion

In this study, the performance of neuromorphic federated learning in the context of HAR was evaluated using two distinct datasets: the UCI dataset and the Real-World dataset. Each dataset offered a unique perspective on the challenges and intricacies of HAR, making them invaluable for a comprehensive assessment. The UCI dataset was divided into 5 participants, and the Real-World dataset was already collected for 15 subjects, which was used to train participants. As discussed earlier, there was an 80–20 split on each participant to obtain the local training and testset. Furthermore, the local testset for each participant was combined to obtain a global testset used to evaluate the generalised performance of the model. The results of each dataset are discussed in the subsequent sections.

### 5.1. UCI Results

**Figure 5:**
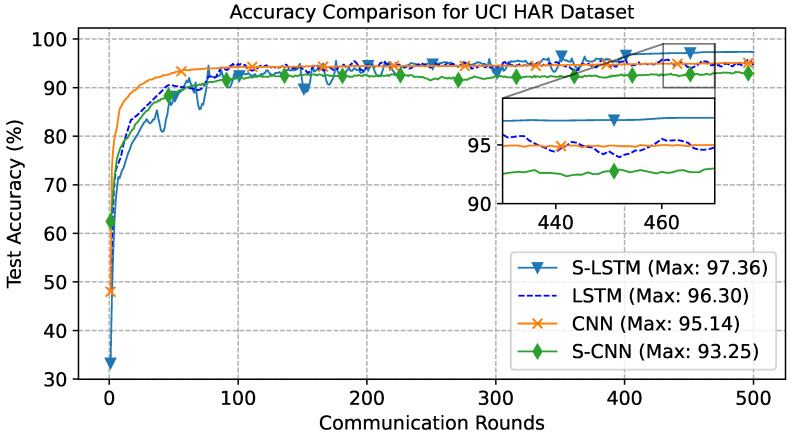
Learning curve for UCI-dataset, trained for 500 communication rounds.

**Figure 6:**
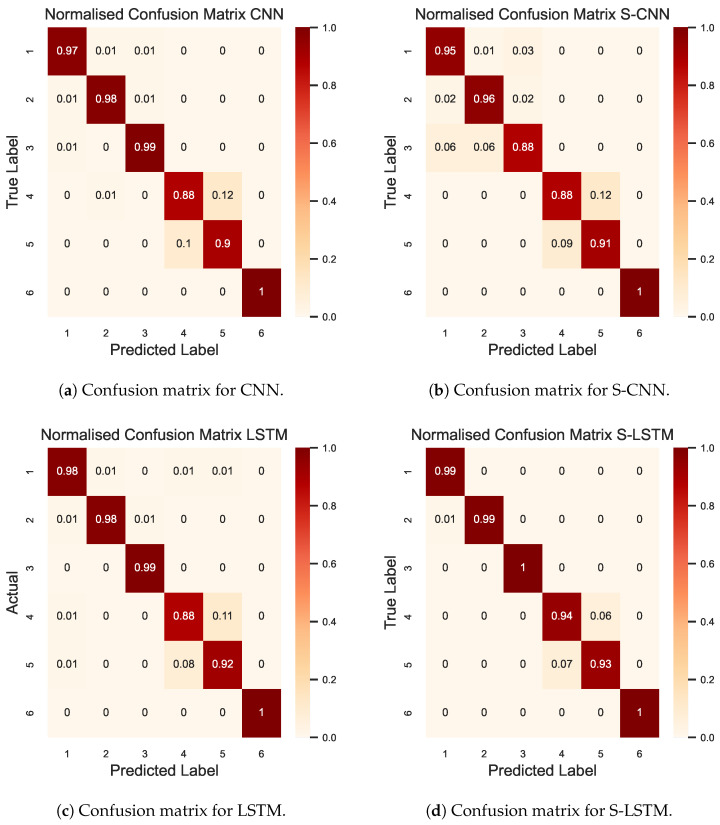
The confusion matrix for four DL models compared in this study. The index represents the activity where the label corresponding to the activities are (1) walking, (2) walking upstairs, (3) walking downstairs, (4) sitting, (5) standing, and (6) lying.

In the UCI case, the total number of communication rounds was kept at 500, where each participant trained the local model for 3 epochs. The results depicted in Figure 5 illustrate the comparison of learning curves for four distinct models, S-LSTM, LSTM, CNN, and S-CNN, analysed within this investigation. These curves exhibit a classical learning behaviour, with a steep rise in the initial training rounds, followed by a steady increasing phase. The S-LSTM model achieved a peak accuracy of 97.36%, demonstrating its superior ability to process and learn from sequential data. The LSTM model followed closely, achieving a peak accuracy of 96.30%. In contrast, the CNN model peaked at 95.14%, and the S-CNN model at 93.25%, indicating a less optimal performance for these architectures in the context of time-series data characteristics of the UCI HAR dataset. The results in Figure 6 depict the normalised confusion matrices for the CNN, S-CNN, LSTM, and S-LSTM models, providing insights into each model’s predictive accuracy across various activities in the UCI HAR dataset. The diagonal elements of the confusion matrix represent the precision of predictions for each class. Notably, in Figure 6d, the confusion matrix of the S-LSTM model demonstrates its ability, with true positive rates exceeding 0.99% for classes 1, 2, and 3. Furthermore, distinguishing between classes 4 (sitting) and 5 (standing) was inherently complex, but the S-LSTM model showed impressive proficiency with a misclassification rate of only 0.06%. This demonstrates its enhanced ability to identify the subtle temporal patterns that differentiate these activities. The CNN and S-CNN models displayed misclassification rates of 0.12% and 0.11%, respectively, for class 4 and class 5, while the LSTM showed a rate of 0.11%. It is worth noting that class 6 (lying) was predicted with perfect accuracy by all models. The true positive rate of 100% was consistently achieved, which can be attributed to the easily distinguishable features of the lying activity in comparison to other activities.

The results in Table 1 present a comprehensive comparative analysis of precision, recall, and F1-score for the four models under consideration when trained on the UCI dataset. The evaluation covered six daily activities to showcase each model’s ability to classify these distinct actions. The results show that the S-LSTM performed better compared to other models with consistently high F1-scores, peaking at 0.99% for both walking and walking upstairs and achieving a perfect score of 1.00 for walking downstairs and lying Each model accurately classified lying because of its distinct motion patterns. Nonetheless, the results also reveal a relative challenge in distinguishing between sitting and standing. While the S-LSTM model exhibited slightly better performance with F1-scores of 0.93% for sitting and 0.94% for standing, the CNN and S-CNN models showed marginally lower F1-scores, ranging from 0.88% to 0.90%. This indicates that the motion patterns for sitting and standing activities are closely related, resulting in difficulty in discerning them, particularly for the CNN and S-CNN models.

### 5.2. Real-World Dataset Results

**Figure 7:**
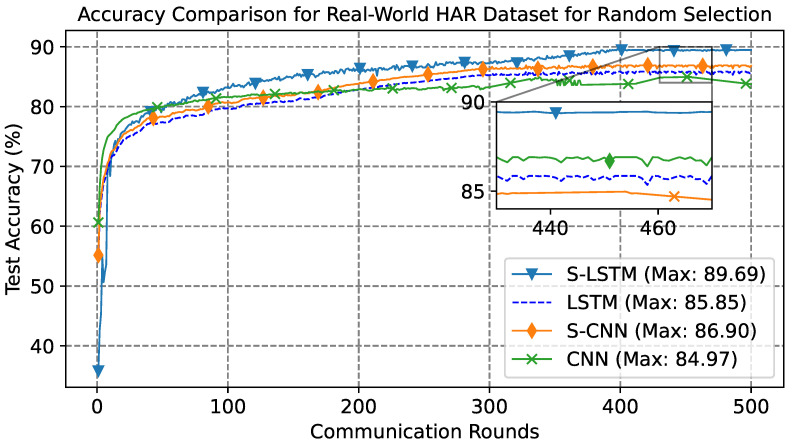
Learning curve for Real-World dataset, trained for 500 communication rounds.

In the case of the Real-World dataset, the experimental setup runs for 500 communication rounds, wherein each participant was trained for five epochs locally. The results in Figure 7 present a comparative learning curve analysis using the Real-World dataset. The learning curves exhibit classical learning, and the results underscore the enhanced capabilities of the spiking models, with both S-LSTM and S-CNN outperforming the conventional LSTM and CNN models. The S-LSTM model achieved an accuracy of 89.69%, while the S-CNN closely follows with an average accuracy of 86.90%. The conventional LSTM and CNN models, on the other hand, attained maximum accuracies of 85.85% and 84.97%, respectively.

**Figure 8:**
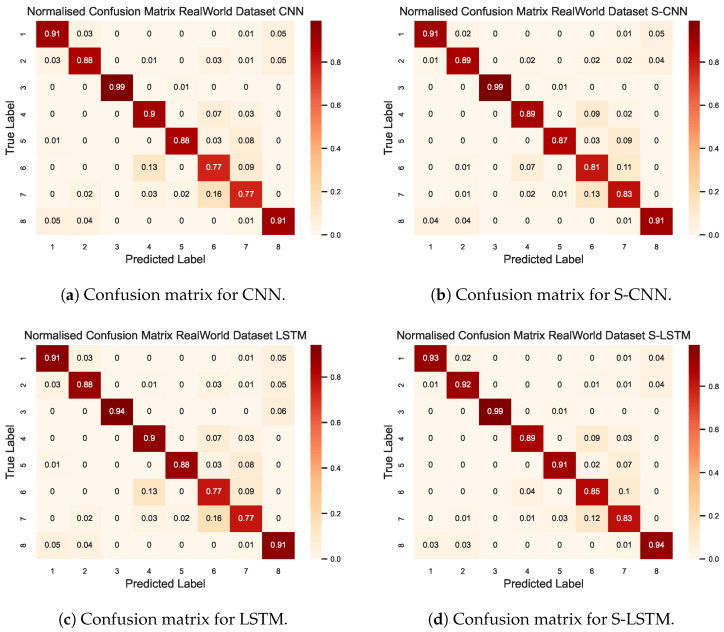
The confusion matrix for four DL models compared for Real-World data set. The index represents the activity where the label corresponding to the activities are: (1) climbing down, (2) climbing up, (3) jumping, (4) lying, (5) running, (6) sitting, (7) standing, (8) walking.

The confusion matrix of the four models considered in this study is illustrated in Figure 8. More specifically, Figure 8d provides a detailed overview of the S-LSTM model’s classification capabilities for eight different activities in the Real-World HAR dataset. The diagonal elements indicate the recall score of the predicted class, where the results show that the S-LSTM model was proficient in classifying more prominent activities, with a score of 0.96% for jumping, 0.86% for running, and 0.91% for walking. However, the confusion matrix also reveals that the S-LSTM model encountered challenges in classifying activities with subtle motion patterns, such as climbing down 0.88%, climbing up 0.87%, and standing 0.80%. Misclassifications mainly arose between activities that share similar motion characteristics. For example, climbing down was occasionally misclassified as walking, climbing up was confused with running, and sitting was sometimes classified as standing.

The results in Table 2 provide a thorough comparison of the models trained on the Real-World HAR dataset in a federated environment. The results show that the S-LSTM model consistently outperformed its counterparts across the spectrum of classification metrics: precision, recall, and F1-score. For example, in the activity walking, S-LSTM achieved a precision of 0.94%, recall of 0.93%, and F1-score of 0.94%, better than the other models considered. Notably, in activities that involve complex motion patterns, such as climbing down and climbing up, the S-LSTM model achieved the highest F1-score of 0.94% and 0.93%, respectively. This indicates its capability to capture and classify intricate temporal patterns more accurately. Additionally, it is worth noting that all models faced challenges in differentiating between sitting and standing, with F1-scores hovering around the 0.73% to 0.85% range, with S-LSTM achieving higher scores compared to others.

**Figure 9:**
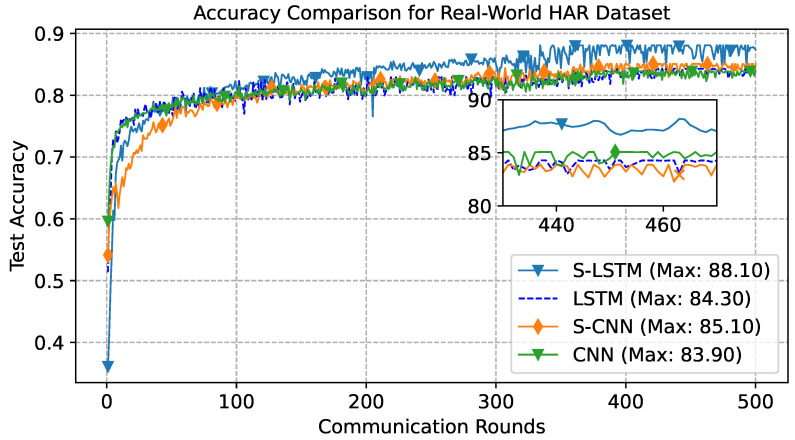
Learning curve for Real-World dataset, with 50% random choosing of participant trained for 500 communication rounds.

To further enhance our comparative study, we adopted a strategy to select 50% of participants randomly for training and aggregation in FL settings. The results in Figure 9 depict the accuracy learning curve, providing comprehensive insight into the performance trajectory over 500 communication rounds. This approach assessed the impact of reduced client participation on model performance and communication cost benefits. From the results, S-LSTM emerges as the top performer, achieving an accuracy of 88.10%. S-CNN follows this with an overall accuracy of 85.10%, LSTM with 84.30%, and the CNN serving as a baseline with 83.90%.

To further enhance our comparison, the energy efficiency models under consideration were rigorously evaluated and quantified as energy estimates defined according to Equation (15). The energy efficiency was calculated in a single communication round with all participants and randomly selected 50% participants. For the purposes of this assessment, the computation constant α and communication constant β were assumed to be 0.003 and 0.0001, respectively, as given in the literature [40]. The results in Table 3 present the energy estimates $E_{est}$, which were contingent upon both the computation time and the model parameter $P_{trn}$, under the specified client selection criterion. Notably, the S-LSTM model had minimal computation time for a single communication round, which stood at 208 s when all participants were included and was further reduced to 121 s with the selection of 50% of participants. Regarding energy consumption, the S-LSTM model demonstrated superior efficiency, registering the lowest energy estimate at 6.10 watts. This result demonstrates a 24.41% decrease in energy requirements compared to the LSTM model, which recorded an energy estimate of 8.07 watts. The results also confirm that using a 50% random participant selection strategy significantly improved the energy efficiency of the model. However, it is important to acknowledge that there is a trade-off between energy efficiency and model accuracy. Finding the right balance between these two aspects is crucial when applying deep learning models, especially when energy conservation is essential.

**Figure 10:**
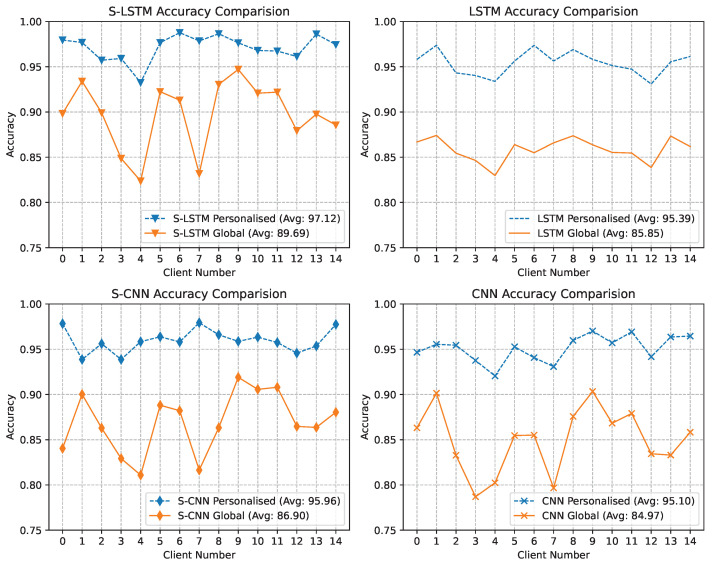
Accuracy comparison graph for global and personalised models for each client using the local test set. The personalised accuracy was obtained after fine-tuning using the local dataset.

All the previous results discussed were obtained using the global model and combined global test set. However, the participant had the processing capabilities to fine-tune the global model to customise it using the local data. Therefore, we also evaluated the performance of the personalised model using the local test set of each participant, and the results are compared with the global model. The results depicted in Figure 10 show the contrast of the test accuracy for global and personalised models at the client level. Spanning 15 clients, the x-axis enumerates each client, with two accuracy plots for each. Personalised accuracy was obtained using the local testset after fine-tuning the global model using the local data. Personalisation improved performance and emphasised the model’s adaptability to specific local data. All four model architectures, including S-LSTM, which boasts the highest global accuracy, exhibit this trend. These findings underscore the advantages of personalisation in FL, where client-specific fine-tuning can substantially improve the local accuracy, which is evident in the results where the average accuracy for S-LSTM improved from 89.69% to 97.12%.

## 6. Conclusions

This paper proposes an HNFL framework that combines the energy-efficient SNN and LSTM network for accurate indoor and outdoor HAR using wearable sensor data. The S-LSTM architecture integrates LSTM layers to capture sequential dependencies in time-series sensor data with spiking layers that provide event-driven processing for energy efficiency. The model was trained using a combination of surrogate gradient learning and BRTT, which enabled supervised end-to-end learning. Extensive evaluations were performed on two publicly HAR datasets – UCI and Real-World – which exhibited distant data distributions and activities. The simulation results demonstrated that the proposed S-LSTM model achieved higher accuracy than LSTM, CNN, and S-CNN models in the federated settings. For instance, S-LSTM showed an improvement of 1.06% compared to the LSTM model for the indoor scenarios. In the case of a more diverse Real-World outdoor dataset, S-LSTM showed an improvement of 3.84% in accuracy compared to simple LSTM. Furthermore, the results also showed a significant improvement in energy efficiency of 32.30%, compared to LSTM. Additionally, random participant selection could significantly improve energy efficiency; however, there was a trade-off between accuracy and efficiency. Additionally, fine-tuning the global model to achieve personalisation improved the performance by 9% on average for each participant.

## Figures and Tables

**Table 1:** Comparative results of global models for CNN, S-CNN, LSTM, and S-LSTM for the UCI dataset trained in federated environment.

	CNN			S-CNN			LSTM			S-LSTM		
**Class**	**Precision**	**Recall**	**F1-Score**	**Precision**	**Recall**	**F1-Score**	**Precision**	**Recall**	**F1-Score**	**Precision**	**Recall**	**F1-Score**
Walking	0.98	0.97	0.98	0.93	0.95	0.94	0.99	0.98	0.99	0.99	0.99	0.99
Walking upstairs	0.98	0.98	0.98	0.93	0.96	0.95	0.99	0.99	0.99	0.99	0.99	0.99
Walking downstairs	0.98	0.99	0.98	0.93	0.88	0.91	0.99	0.99	0.99	1.00	1.00	1.00
Sitting	0.89	0.88	0.88	0.90	0.88	0.89	0.91	0.90	0.91	0.93	0.94	0.93
Standing	0.89	0.90	0.89	0.89	0.91	0.90	0.91	0.92	0.91	0.94	0.93	0.94
Lying	1.00	1.00	1.00	1.00	1.00	1.00	1.00	1.00	1.00	1.00	1.00	1.00

**Table 2:** Comparison of classification metrics between different DL techniques for Real-World dataset.

	CNN			S-CNN			LSTM			S-LSTM		
**Class**	**Precision**	**Recall**	**F1-Score**	**Precision**	**Recall**	**F1-Score**	**Precision**	**Recall**	**F1-Score**	**Precision**	**Recall**	**F1-Score**
Climb down	0.90	0.91	0.90	0.92	0.91	0.91	0.90	0.91	0.90	0.94	0.93	0.94
Climb up	0.90	0.88	0.89	0.92	0.89	0.90	0.90	0.88	0.89	0.93	0.92	0.93
Jumping	1.00	1.00	1.00	1.00	1.00	1.00	0.99	0.94	0.96	1.00	1.00	1.00
Lying	0.84	0.90	0.87	0.89	0.89	0.89	0.84	0.90	0.87	0.95	0.89	0.92
Running	0.98	0.88	0.93	0.98	0.87	0.93	0.98	0.88	0.93	0.97	0.91	0.94
Sitting	0.73	0.77	0.75	0.74	0.81	0.77	0.73	0.77	0.75	0.78	0.85	0.82
Standing	0.77	0.77	0.77	0.75	0.83	0.79	0.77	0.77	0.77	0.79	0.83	0.81
Waling	0.91	0.91	0.91	0.92	0.91	0.91	0.91	0.91	0.91	0.93	0.94	0.93

**Table 3:** Comparison of energy efficiency of the proposed hybrid model using the metric energy estimates given in Equation (15). In this case, the 50% random participant selection was made, and the energy estimate was calculated for one communication round.

Model	Model Parameter $P_{trn}$ (KB)	Computation Time $t_{com}$ (s)	Energy Estimate $E_{est}$ (W)
CNN	25321	258	38.24
		143	15.73
S-CNN	19418	252	29.38
		136	15.67
LSTM	5231	220	8.07
		137	4.32
S-LSTM	3931	208	6.10
		121	3.27

## Data Availability

This study is conducted using a publicly available datasets. Here are the links of the datasets.
